# Insights of private general practitioners in group practice on the introduction of National Health Insurance in South Africa

**DOI:** 10.4102/phcfm.v8i1.1025

**Published:** 2016-06-15

**Authors:** Shabir Moosa, John Luiz, Teresa Carmichael, Wim Peersman, Anselme Derese

**Affiliations:** 1 Department of Family Medicine, University of Witwatersrand, South Africa; 2 Graduate School of Business, University of Cape Town, South Africa; 3 Wits Business School, University of Witwatersrand, South Africa; 4 Department of Family Medicine, Ghent University, Belgium

## Abstract

**Background:**

The South African government intends to contract with ‘accredited provider groups’ for capitated primary care under National Health Insurance (NHI). South African solo general practitioners (GPs) are unhappy with group practice. There is no clarity on the views of GPs in group practice on contracting to the NHI.

**Objectives:**

To describe the demographic and practice profile of GPs in group practice in South Africa, and evaluate their views on NHI, compared to solo GPs.

**Methods:**

This was a descriptive survey. The population of 8721 private GPs in South Africa with emails available were emailed an online questionnaire. Descriptive statistical analyses and thematic content analysis were conducted.

**Results:**

In all, 819 GPs responded (568 solo GPs and 251 GPs in groups). The results are focused on group GPs. GPs in groups have a different demographic practice profile compared to solo GPs. GPs in groups expected R4.86 million ($0.41 million) for a hypothetical NHI proposal of comprehensive primary healthcare (excluding medicines and investigations) to a practice population of 10 000 people. GPs planned a clinical team of 8 to 12 (including nurses) and 4 to 6 administrative staff. GPs in group practices saw three major risks: patient, organisational and government, with three related risk management strategies.

**Conclusions:**

GPs can competitively contract with NHI, although there are concerns. NHI contracting should not be limited to groups. All GPs embraced strong teamwork, including using nurses more effectively. This aligns well with the emergence of family medicine in Africa.

## Background

Healthcare in South Africa (SA) is fragmented and inequitable, with public health funds servicing 85% of the population, whilst a similar amount is spent privately by 15% of the population on voluntary prepaid medical insurance. Private costs are escalating, driven by hospitals and specialists.^1^ Most generalist doctors are in private practice, mostly fee-for-service, and function only with undergraduate training. The public primary healthcare (PHC) system is ‘nurse-driven’ with doctors in a marginal role, ostensibly because of shortages of doctors and a GP bias against early post-apartheid public health reforms.^2,3^ Full-time postgraduate training in family medicine only started in 2008 with family physicians focused on public service district hospitals. Key stakeholders in SA see family physicians as critical to the district health services (DHS), with a growing focus on team-based family practices and community-oriented primary care.^4^ Private general practitioners (GPs) do provide some services to the public service, with sessions (where they work in clinics and are paid per hour), or offer free immunisation, family planning and HIV counselling and testing in their rooms with free materials provided by the public service, and an informal arrangement that only services are charged for and not materials.

The South African government is planning a National Health Insurance (NHI) system from 2012 to 2025 to address the public-private inequity in spending and human resource and to harness private resources, including GPs. GPs are to be included in capitation contracts alongside current nurse-dominated public PHC services.^5^ The views of GPs working in group practices are important as the NHI expects capitation contracts to be with accredited provider groups.^5^ The uptake in capitation contracts depends on rates being acceptable to providers.^6^ The major cost elements are staff, operations and investments.

Currently, utilisation of the public DHS in SA is approximately two visits per person per year, although it is expected to be three under NHI, because of improved services. In NHI policy debate in SA, it has been raised that any random pool of 1000 people (as the smallest number) would share the same actuarial risks for primary care and any random pool of 20 000 people (as the smallest number) would share the same actuarial risks for hospitalisation, depending on rurality.^7^ A risk pool and potential practice list of 10 000 can allow GPs to explore team-based care, based on South African experience of task shifting.^8^ This number is also based on World Health Organization’s use of 10 000 as a population denominator for one medical practitioner.^6^ A utilisation rate of three visits per person per annum (50% more than current PHC service in SA) would yield 120 visits per day, with half the visits for personal-curative services (provided by doctor/PHC nurses) and the other half for preventive-promotive services (provided by other nurses).^9^ We examined solo GPs in SA in an earlier paper based on this proposal.^8^ In this article, we describe the demographic and practice profile of GPs in group practice, evaluate their views of NHI and their responses to this NHI proposal and compare it to GPs in solo practice.

## Methods

### Study design

This was a descriptive online study using a self-administered questionnaire.^10^

### Setting

The study population consisted of 8721 private GPs (both solo and in group practice) licensed by the Board of Healthcare Funders to practice privately in SA, and with emails available

### Sampling and selection

Questionnaires were sent to all 8721 private GPs (solo and in group practice).

### Data collection

A personalised email and questionnaire were sent to these GPs during April 2011. Email addresses were corrected by SMS prior to the survey, with two email reminders and three SMS reminders over a period of 3 weeks to follow-up with non-responders. The questionnaire had three sections: demographic characteristics, practice, and a section exploring their response to a hypothetical NHI contract for the provision of personal-curative and preventive-promotive healthcare (excluding medicines and investigations) to a practice population of 10 000, based on an utilisation of three visits per person per year. The assumptions outlined were that ± 60 patients would visit daily for personal-curative and that ± 60 patients would visit daily for preventive-promotive healthcare. GPs were asked to provide current and expected costs and to state the minimum global fee they would accept for such a hypothetical contract. Open-ended written questions explored the risks they perceived and their risk management strategies for them. The questionnaire was tested with a set of six GPs and qualitative feedback on construct and content validity was obtained. There was marginal change.

### Data analysis

Quantitative data analysis was carried out with SPSS (IBM Corp. Released 2013. IBM SPSS Statistics for Macintosh, Version 22.0. Armonk, NY: IBM Corp). Numeric data were analysed using frequency tables and histograms. Those with normal distributions were tested for statistical difference using independent-samples *t*-tests. Those with non-normal distributions were tested for statistical differences using the Mann-Whitney test. Categorical data were analysed using cross-tabulations with tests for statistical difference using Pearson chi-square tests. A *P*-value < 0.05 was considered statistically significant. Qualitative data, as short written responses to two open-ended questions, were examined using colour coding on MS Excel for themes in thematic content analysis. This thematic analysis was validated by other authors and finalised for presentation.

## Ethical approval and funding

Wits Business School provided ethical approval on 22 March 2011. Respondents were not offered monetary reward for participation. The data produced remain confidential, with respondents anonymous in all analyses. The study received part funding from the European Union’s Seventh Framework Programme (FP7-AFRICA-2010) under grant agreement no. 265727.

## Results

There were a total of 819 respondents overall. These GPs were asked to classify themselves in terms of solo or group practice. Whilst the focus of the article is on the 251 GPs from group practice, the responses of the 568 solo GPs have been added for easy comparison (albeit with small corrections of previously published data,^8^ based on removing 30 responses that had less than 50% of the questionnaire completed).

Demographic and practice profiling (Table 1) show that GPs in groups are significantly younger than those in solo practice. They work for fewer days in the month and see more patients per day. They also have significantly higher consultation fees at R262.40 ($20.20) versus R236.90 ($18.24) for solo GPs (based on an exchange rate of R1.00 = $0.077).

**TABLE 1:** Demographics and practice profile.

Practice profile	Group (*n* = 251)			Solo (*n* = 568)		
	
	Mean	(s.d.)	*n*	Mean	(s.d.)	*n*
Age in years	43.0	(9.9)	226	45.9	(10.9)	536
Experience in years	14.9	(9.8)	232	16.5	(11.1)	533
Days working per month	23.4	(3.1)	233	24.1	(2.6)	531
Number of patients seen daily	28.6	(14.1)	233	22.6	(12.2)	535
Medical Aid Scheme patients as % of total	62.4	(21.4)	236	59.6	(24.6)	554
Consultation fee in rand	262.4	(69.2)	231	236.9	(66.2)	531

Further demographic profiling (Table 2) shows that there are significantly more female and white GPs in groups than male GPs and black people (inclusive of African, Indian and mixed race, as per apartheid classifications). GPs in groups are significantly fewer in townships compared to solo GPs, although their spread across various provinces is not significantly different to solo GPs. Further practice profiling (Table 2) shows two significant differences: that GPs in groups are more optimistic on future practice and that they use computers more in their practice, compared to solo GPs. Otherwise, current practice, and engagement with government contracting, capitation and NHI are not significantly different.

**TABLE 2:** Demographics and practice profile.

Variables	Group (*n* = 251)		Solo (*n* = 568)	
	
	%	*n*	%	*n*
**Gender**				
Male	28.3	166	71.7	421
Female	35.4	80	64.6	146
**Race**				
White	42.0	163	58.0	225
Black (incl. black, indian and coloured)	19.3	81	80.7	339
**Province**				
Urban (Gauteng/Western Cape)	33.9	140	66.1	273
Big Rural (Eastern Cape/KwaZulu Natal	25.2	52	74.8	154
Small rural (Free State / Limpopo/Northern Cape/North West)	27.8	52	72.2	140
**Area**				
Towns/Rural other	31.3	86	68.7	189
Townships	13.6	15	86.4	95
City centre/Suburbs	33.8	144	66.2	282
**Practice growth**				
Practice grown last 5 years	90.5	210	86.0	474
Practice expected to grow next 5 years	91.5	215	83.2	460
**Engagement with government contracts**				
Doing Sessional Contracts	20.7	46	26.9	141
Providing Immunisation	11.1	22	8.7	38
Providing Family Planning	17.1	35	21.2	96
Providing HIV Counselling/Testing	18.6	38	23.0	105
Doing Other contracts	12.3	22	14.1	54
**Engagement with capitation**				
Using computers in practice	53.2	124	41.7	227
Reviewing data in practice	60.5	138	60.4	329
Capitation understanding is good (vs. poor)	77.7	185	75.6	419
Capitation patients > 20% of patient base	25.1	56	25.0	132
Supportive of NHI	39.0	90	32.5	178

GPs described their current staffing as well as additional staffing for the NHI proposal (Table 3). GPs in groups currently use nurses more than solo GPs. GPs in groups added significantly more professional and PHC-trained nurses under the NHI proposal. GPs in groups feel that they would need to almost double the number of staffing, especially nurses. Both solo and group GPs added two administrative staff to current staff. GPs also described their mean practice costs, both current and future, with suggestions for a global fee for the NHI proposal. GPs in groups currently have significantly higher staffing and operational expenditure as a percentage of their practice expenditure, compared to solo GPs. However, GPs in groups expect to incur significantly less staffing and operational and investment costs than solo GPs. The global fee is significantly higher for GPs in groups at R4.86 million ($0.37 million) compared to solo GPs at R4.07 million ($0.31 million) (based on an exchange rate of R1 = $0.077).

**TABLE 3:** Practice management and response to National Health Insurance Contract.

Variables	Group (*n* = 251)			Solo (*n* = 568)		
	
	Mean	(s.d.)	*n*	Mean	(s.d.)	*n*
**Current staff**						
Nursing assistant	1.1	(1.5)	135	0.6	(0.9)	316
Staff nurse	0.9	(1.1)	136	0.2	(0.4)	230
Professional nurse	1.5	(1.8)	150	0.3	(0.6)	254
PHC-trained nurse	0.4	(1.0)	95	0.1	(0.4)	215
Doctor	2.7	(1.8)	190	0.7	(0.7)	303
Administrative	3.8	(2.1)	212	1.9	(1.1)	492
**Additional Staff for NHI scenario**						
Nursing assistant	1.5	(1.6)	120	1.2	(0.9)	270
Staff nurse	1.4	(1.3)	131	1.1	(0.7)	245
Professional nurse	1.5	(1.3)	140	1.0	(0.7)	270
PHC-trained nurse	1.6	(1.4)	135	1.1	(0.7)	318
Doctor	1.5	(1.4)	127	1.2	(0.8)	273
Administrative	2.0	(1.7)	159	1.8	(1.1)	364
**Current costs**						
Practice (as % of turnover)	48.6	(16.3)	186	46.8	(19.3)	477
Staff (as % of practice expenditure)	36.6	(20.1)	185	28.1	(20.4)	477
Operations (as % of practice expenditure)	32.5	(19.4)	181	29.4	(20.3)	474
**Expected costs for NHI scenario**						
Staff (as % additional to current expenditure, up to > 200%)	58.9	(49.2)	185	88.1	(68.2)	463
Operations (as % additional to current expenditure, up to > 200%)	42.4	(43.6)	183	57.6	(58.9)	461
Investment (as % additional, up to > 200%)	40.0	(46.0)	183	58.1	(59.5)	460
**Global fee**						
Global fee (in R millions)	4.86	(3.0)	182	4.07	(2.7)	447

**FIGURE 1:**
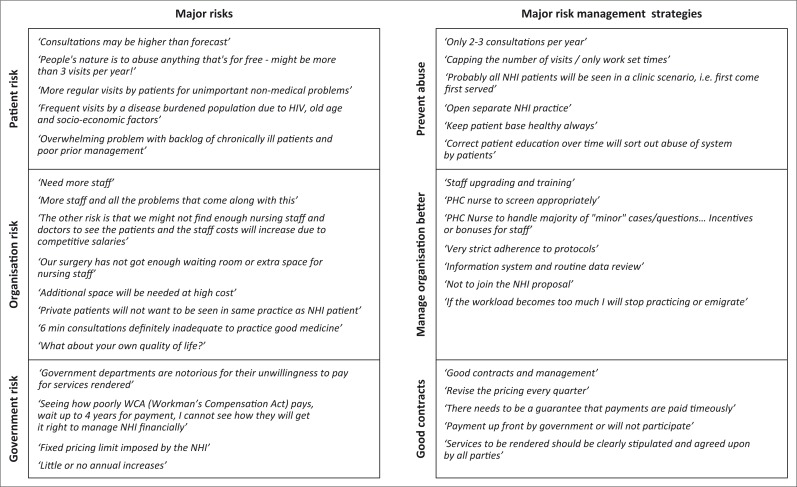
Risks and risk management strategies by group practice General Practitioners.

In the open-ended questions the 167 GPs in groups felt that there were three major risks in contracting for NHI: organisational, patient and government and thus proposed three key strategies to manage their risks in contracting for NHI: organisational management, preventing patient abuse and good contracts (Figure 1).

The key risk GPs in groups saw coming from patients was over-utilisation from abuse and need. They felt that their services would be abused, with unnecessary visits ‘People’s nature is to abuse anything that’s for free − might be more than 3 visits per year!’ They suggested a rate of above five per year based on their experience in the DHS (with heavy disease burden of HIV, tuberculosis, multiple chronic diseases and age profile) and their experience of capitation patients in managed healthcare.

The resulting risk management strategy for GPs in groups was to control patient numbers. This was either to cap the numbers of visits to ‘only 2 to 3 consultations per year’, see patients on a first come first served basis, working at fixed times or opening a separate NHI practice as a clinic. There was a minor view to educate patients, engage in preventive care and keep patients healthy.

The key risks GPs in groups saw for their organisation were staff and space. A large number of respondents felt that the NHI proposal would mean ’more staff, and all the problems that come along with this’. They also needed more space. They thought that ‘additional space will be needed at high cost’ with expansion for waiting rooms, consulting, radiology, procedures, dispensing etc.; adjustments (like ventilation) and wear and tear. More than half were concerned about the impact on their current patients: ‘Private patients will not want to be seen in the same practice as NHI patient’.

The resulting risk management strategy for GPs in groups was to address their organisational structure, especially staff organisation and ‘staff upgrading and training’. This included screening procedures, protocols, data review and audits. The PHC nurse was expected to feature strongly in the NHI team ‘PHC nurse to handle majority of ”minor“ cases/questions’. They also felt that they needed to develop better practice management. A few said they would opt out and focus on private patients, stop practicing or emigrate.

The key risk GPs in groups saw coming from government, was government abusing the contract through poor payment, poor pricing and unclear service packages. Their big concern was whether government would pay them on time and how they would manage their cash flow. They felt government to be corrupt, unreliable and ‘notorious for their unwillingness to pay for services rendered’ and that they would suffer a ‘fixed pricing limit(s) imposed by the NHI’. They wanted pricing to be explicit for a number of variables: medicines, pathology, radiology, procedures, preventive care, professional time in meetings, competition, eligibility, morbidity profile, and so on.

The key risk management strategy for GPs in groups was to ensure a strong contract with government. Respondents felt that there needed to be consultation and negotiation on conditions by GP bodies, good communication from government, lump-sum upfront payments, clear contracts, phased approaches, periodic adjustments and escape clauses, for example one GP said: ‘Payment up front by government or [*we*] will not participate.’

## Discussion

GPs in group practices have more younger people, more white people, more women and operate more in city suburbs and small towns or rural towns when compared to solo GPs. They work for fewer days, have more patients per day, a higher consultation fee and are more optimistic about the future versus GPs in solo practice. Otherwise, practice and NHI support appears to be not significantly different. GPs in groups wanted more staff but added less expenditure for staff, operations and investments than solo GPs did for the NHI capitation contract proposal. The global fee per year for GPs in groups was significantly higher at R4.86 million ($0.41 million) compared to solo GPs at R4.07 million ($0.34 million). GPs in group practices saw three major risks in contracting for NHI: patient, organisational and government, with three related risk management strategies: better management, preventing patient abuse by controlling numbers and ensuring strong contracts with government.

Plans for NHI speak of contracting with ‘accredited provider groups’,^5^ but GPs in groups appear as equivocal in their support for NHI as solo GPs but more optimistic about the private sector compared to solo GPs, with better patient numbers and consultation fees.^2^ Younger white female doctors seem attracted to groups, as practice becomes complex and groups accommodate their life choices.^11,12^ GP loss is a possibility with NHI ‘imposed’ on an ageing GP population. Poor working conditions and a poor career path drive brain drain.^13^ NHI capitation contracts with GPs offer a way to harness this resource and support a nurse-driven PHC system that is overwhelmed.^3^

Whilst the group GPs global fees for the capitation proposal are significantly higher than that of solo GPs, it, is still competitive with current public expenditure.^8^ Many patients prefer private GPs, spending almost half of the national out-of-pocket expenditure (equal to ± 30% of the national public health budget) on GPs in 2009.^14^ Black solo GPs appear to be having poor practice conditions whilst working more in townships and may be more useful and more amenable to contracting with the NHI, if their concerns are addressed.

Contracting GP-led teams can improve professional behaviours, health system and outcomes,^15,16^ but it is important to address GP perceptions of uncontrolled risk with capitation.^17^ Capitation models with accountability for outcomes appear in most policy initiatives in United States, United Kingdom and Canada.^11,18,19^ The current risk management strategy of GPs in groups showed moral hazard by limiting access, as compared to the risk management strategy of solo GPs to promote health.^8,20,21^ There are potential perversities with GPs setting up separate NHI practices. This harks back to difficult apartheid days when district surgeons serviced white patients at the front and black patients at the back.^22^ On the other hand, there is genuine fear with little information in SA on fully capitated population risk.^23,24^ GPs were speculating that utilisation would be 8 to 10 visits per person per annum. By comparison, utilisation in the UK is five.^25^ Their limited exposure to capitation and population-level behaviour leaves the fee-for-service practice mindset intact.^15,23^

Staff numbers doubled for group GPs versus solo GPs, with extensive use of nurses in a clinical team of 8 to 14 members for a practice population of 10 000. This is very encouraging for efficient practice based on task shifting but needs triangulation using Workload Indicators for Staffing Needs.^26^ Group GPs cost increases were less than those of solo GPs, suggesting that GPs in groups were also not overly familiar with their cost structures and require practice management support.

There is great potential to engage GPs in SA, especially solo GPs, with fully capitated contracts, but this seems to be on the backburner^27^ despite solutions proffered to government.^28^ Providing PHC for the 52 million people of SA would require 5200 GPs and cost R27.2 billion ($2.09 billion) (based on the GP group fee). This contrasts strongly against NHI starting estimates of R128 billion ($9.9 billion)^29^ and R176 billion ($13.5 billion) for ‘basic primary care’, seemingly based on current fee-for-service costs.^30,31^

GP practice, their engagement with NHI and possible human resource modelling must be examined and tested for costs and quality. Quality care by GPs is possible in SA^32^ despite problems^33^; however, thought needs to be given to the PHC teams’ skills hierarchy to ensure accountability, with reward and responsibility built into provider markets.^28,34^ Risks, and appropriate management of these risks, need to be studied on the path to contracting with GPs under NHI in SA.^28^ Developing larger numbers of GPs through postgraduate training in family medicine is a useful opportunity for the NHI.^35^ The South African government needs to clarify its stance on contracting with GPs and explore innovative solutions from amongst GPs.^8,28^ The emerging role of family medicine in SA and Africa can help guide this project.^3,4,8,28,36,37^

The strength of this study lies in its national reach. Threats to validity include self-selection bias by respondents’ interest and only GPs with emails being included. The low response rate and assumptions on risk pools and utilisation in the NHI proposal were also limitations. There is also limited information to support a practice list of 10 000, apart from African experience in team-based task shifting in PHC.^10,38^ The study is limited by the veracity of arguments for NHI,^5^ a key reference point for assumptions in this study. Generalisation of the results to SA should be carried out carefully, but the study offers insights into the views of GPs working in group practice in SA.

## Conclusion

Whilst much of the global primary care system is based on solo doctors delivering first contact care, there is an international imperative towards team-based care (including nurses, family physicians and community health workers).^39^ GPs in groups and solo practice in SA can competitively contract in the NHI, although population risk and risk management are of concern. NHI contracting should not be limited to groups. All GPs embraced strong teamwork, including using nurses more effectively. This aligns well with the emergence of family medicine in Africa. NHI capitated models of service delivery in SA, based on integrated PHC teams led by GPs, may offer lessons globally for universal health coverage in poorly resourced settings with robust private sectors, and needs to be further evaluated.
